# Frontal changes in medium-sized glaciers in Sikkim, India during 1988–2018: Insights for glacier-climate synthesis over the Himalaya

**DOI:** 10.1016/j.isci.2023.107789

**Published:** 2023-08-30

**Authors:** Parvendra Kumar, Milap Chand Sharma

**Affiliations:** 1 Department of General & Applied Geography, Dr. Harisingh Gour Vishwavidyalaya (A Central University), Sagar, Madhya Pradesh 470003, India; 2 Centre for the Study of Regional Development, Jawaharlal Nehru University, New Delhi 110067, India

**Keywords:** Earth sciences, Earth-surface processes, Glacial processes, Global change

## Abstract

The study assesses terminus retreat of medium-sized glaciers (1988–2018) using geospatial dataset and field study in Sikkim which is under the direct influence of the Indian SW monsoon. It also explores the causes of intra-regional and inter-regional diverse patterns of glacier retreat under the purview of topographical and climatic factors to develop a glacier-climate synthesis over the region. Glaciers have retreated in a range from 63.9 to 3.9 m yr^−1^ and lost a total area of ∼2.53% (0.08% yr^−1^) in the study area. The intra-regional heterogeneity in glaciers retreat seems to be caused by topographical factors in the study area. A comparison of glacier retreats with other parts of the Himalayas reveals a declining gradient from the northwest to the eastern Himalayas, broadly. This inter-regional disparity in the retreat rate seems to be caused by existing climatic regimes over different parts of the Himalayas. The results help to comprehend the glacier-climate synthesis over the Himalayan region.

## Highlights

• New and updated data on the retreat of medium-sized glaciers in Sikkim is reported
• The study presents the glacier-climate synthesis over the Himalaya
• A glacier retreat gradient with its climatological causes in the Himalaya is recorded

## Graphical abstract

**Figure:**
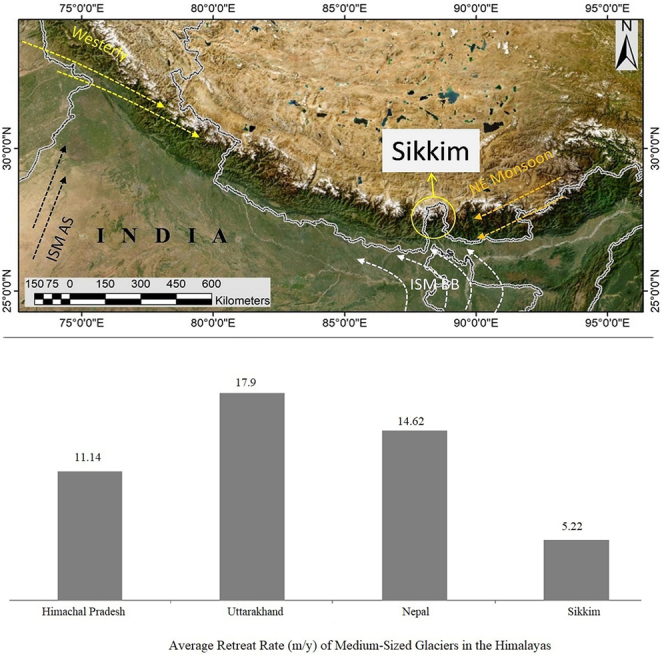

## Introduction

Glaciers are one of the major geomorphic landscapes of the Himalayan region. The Himalayas, constituting the most extensive glacier cover outside the Arctic, and Antarctica is also termed as the Third Pole.^1^^,^^2^^,^^3^^,^^4^ The assessment of existing snow and glaciers in the region is of immense importance from the perspective of water resources, glaciological hazards, and as important indicators of climatic change.^5^^,^^6^^,^^7^^,^^8^^,^^9^^,^^10^ Being located under the direct influence of the Indian monsoon and western disturbance, the Himalayan glaciers provide a reasonable opportunity to understand the climatic change in the region.^11^ Considering the importance of Himalayan glaciers, several studies have attempted to understand the glacier dynamics of the region in the recent past and have reported that the glaciers of the region are in a general state of retreat since 1850 under the influence of reported climatic change.^2^^,^^9^^,^^12^^,^^13^^,^^14^^,^^15^^,^^16^^,^^17^^,^^18^ The accelerated rate of glacier retreat also has been reported in some of the regions of the Himalayas during recent decades.^19^^,^^20^^,^^21^ However, at the same time, glaciers in some regions of the Himalayas have demonstrated a heterogeneous pattern of fluctuation with stagnant or advancing terminus.^22^^,^^23^^,^^24^ Several studies have been carried out to understand the dynamics of the glaciers in the northern, western, and central Himalayas.^14^^,^^15^^,^^18^^,^^19^^,^^21^^,^^24^^,^^25^^,^^26^^,^^27^^,^^28^^,^^29^^,^^30^^,^^31^ However, parts of the eastern Himalayas are still underrepresented in glacier change studies as only a few studies have been carried out. Changes in the glacier processes of this region may have a substantial impact on the drinking water supply and regional water balance along with negative impacts on the agriculture and tourism sectors which support the economy of the region. Glacier hazards such as glacial lake outburst floods are also major concerns as several glacial lakes have emerged in recent decades in this region under the current scenario of climate change.^32^ Therefore, the present study is focused to understand the dynamics of glaciers in the Teesta river basin, Sikkim which consists of an important region of the eastern Himalayas.

Among the few studies of the region, one of the studies has reported on the influence of debris cover on the recession of glaciers (areal changes) from 1989/90 to 2010 and highlighted an accelerated recession of glaciers after 1997 in the region.^33^ A similar study on areal changes in glaciers has reported about 20.1% loss in glacier area from 1962 to 2000 in Sikkim area.^34^ Particularly, detailed field based studies are not available for this region. Further, only limited studies have reported on the length fluctuation at the terminus of glaciers in this region.^35^^,^^36^ Moreover, the findings of most of these studies are based on the glaciers of heterogeneous sizes, which have provided a mixed indication of long as well as short-term climatic impacts, depending on the response time of the glaciers. However, the reporting of different-sized glaciers is suggested to be done separately as smaller glaciers occupy lesser area but retreat faster than larger glaciers and vice versa^15^^,^^18^^,^^20^^,^^37^ due to the diverse response time of smaller and larger glaciers. On the basis of size, small, medium, and large glaciers reflect climate change of less than decadal, decadal, and secular timescales, respectively.^35^^,^^38^^,^^39^ Considering these complexities of glacier-climate interaction, the present study is focused only on medium-sized glaciers to understand the climate-glacier interaction at a decadal scale.

The study aims to assess the frontal changes in length and area of medium-sized glaciers (1988–2018) to develop a comprehensive understanding of the dynamics of glaciers in the region. In addition, the present study also attempts to assess the role of local physiographic factors such as glaciers’ length, size, form, aspect, existence of glacial lakes, altitude of the terminus, and slope gradient of the glaciers to understand the heterogeneous pattern of retreat. Additionally, to develop a glacier-climate synthesis over the region, the study compares the retreat rate of glaciers in Sikkim with other parts across the Himalayas which are under diverse climatic regimes.

### Study area

**Figure 1:**
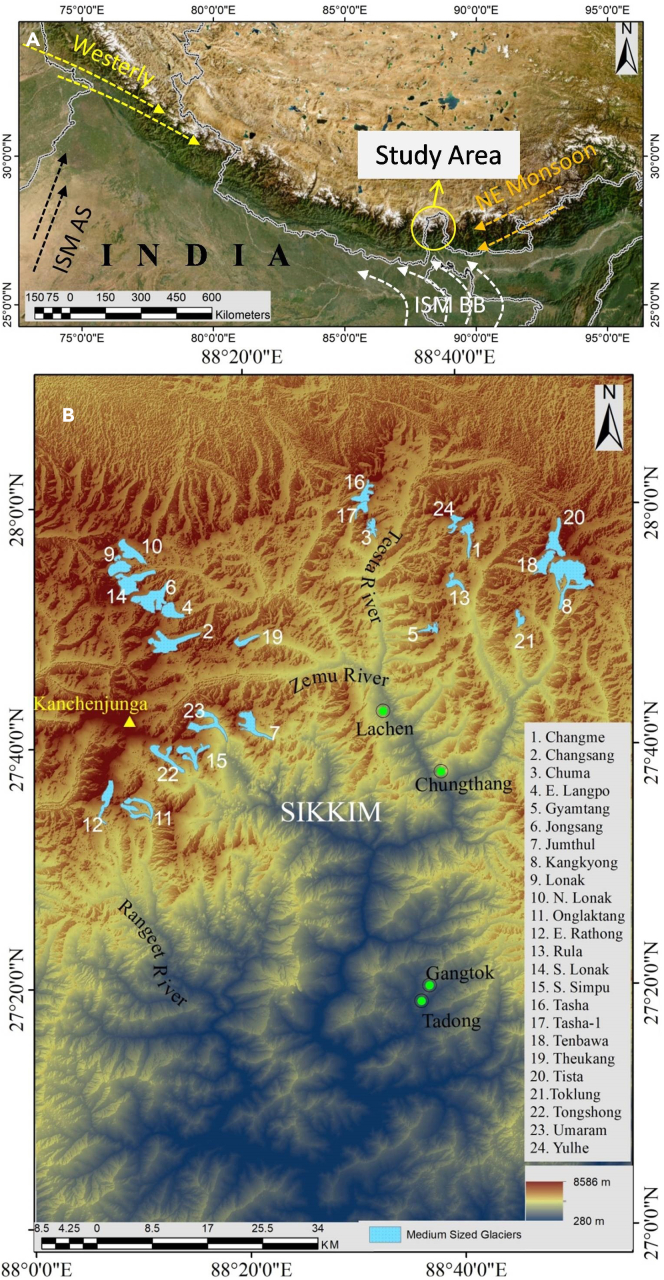
Location of the study area (A) Himalayas with marked location of Sikkim along with sources of precipitation, (B) Medium-sized glaciers in Sikkim. The background image in (B) is a Digital Elevation Model (ASTER GDEM V2) of Sikkim. ISM, Indian Summer Monsoon; AS, Arabian Sea branch; ISM BB, Bay of Bengal branch of ISM.

Sikkim is a part of the eastern Himalaya and consists of the upper Teesta river basin with an area of 7096 km^2^. It extends ∼114 km from North to South and ∼64 km from East to West between 27°00′46″–28°07′48″ N and 88°00′58″–88°55′25″ E. Sikkim has Chola range on the East boundary with Bhutan, Greater Himalaya along with Chola range on the north and northeast boundary with Tibet, and Singalila range on the west boundary with Nepal which makes it a vantage region to trap the incoming moisture-laden winds. The altitude varies between 280 and 8586 m asl. in south to north transects. Sikkim, being located in the eastern Himalaya, receives precipitation mainly from the SW monsoon (Figure 1). In addition, mid-latitude westerlies and Northeast monsoon also contribute in the form of winter precipitation.^40^ The orographic characteristics of the region control atmospheric processes. Months from June to August record maximum temperature while the minimum is observed from December to January with snowfall.^41^ Most of the glaciers exist in the North district of Sikkim. These glaciers feed two major river systems i.e., Teesta River and Rangeet River.

Glacier processes occupied an area of 365.89 km^2^ with more than 90 glaciers in Sikkim Himalaya.^42^ The selected 24 glaciers for the present study are the main medium-sized glaciers and are well demarcated in the Swiss toposheet with names (Figure 1B). These medium-sized glaciers have a length of ∼2–10 km, an area of ∼2–20 km^2^, and share ∼155.20 km^2^ (42.41%) of the total glacierized area. Other glaciers are either smaller or bigger in length and size or are not suitable for the present study due to the difficulty in the demarcation of accurate snout location from the medium spatial resolution images.

## Results

### Glacier characteristics

**Figure 2:**
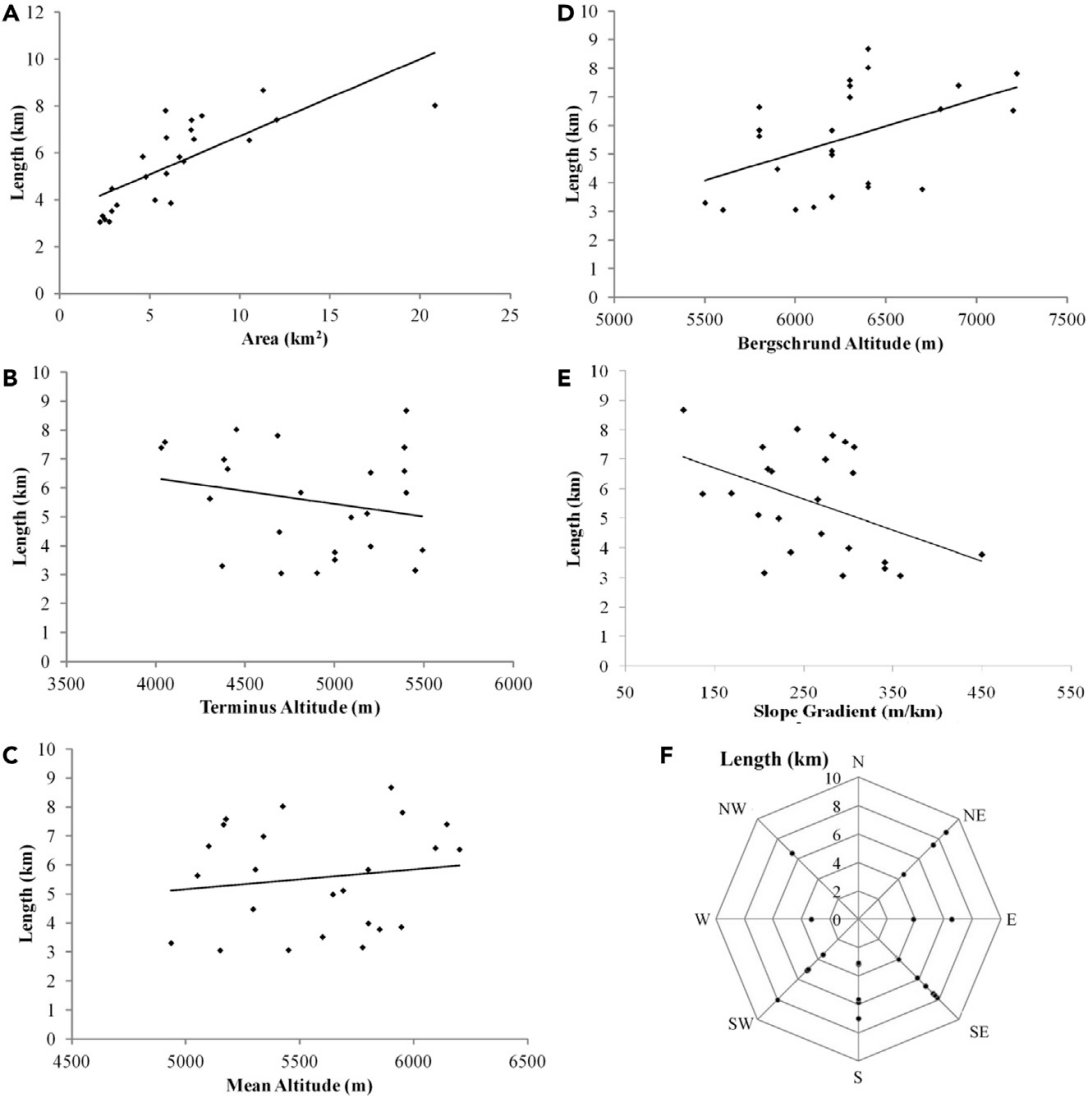
Relationship of glacier’s length with other parameters Glacier length vs (A) Glacier area, (B) Terminus altitude, (C) Mean altitude, (D) Bergschrund altitude, (E) Slope gradient, (F) Ablation orientation.

The size of the 24 selected medium-sized glaciers ranges from 2.2 (Toklung Glacier) to 20.8 km^2^ (Kangkyong Glacier) with a median size of 5.9 km^2^. The 10 glaciers are smaller than the median size and share 33.4 km^2^ (21.52%) area whereas the rest 14 glaciers share 121.9 km^2^ (78.48%) area. The length of the glaciers ranges between 8.7 (Changsang Glacier) and 3.1 km (Toklung Glacier) with a median of 5.7 km which divides the total glaciers into two-halves. The glaciers shorter than the median length share ∼47.7 km^2^ (30.74%) area while the glaciers longer than the median length share ∼107.5 km^2^ (69.26%) area of the total area of the assessed glaciers. It is apparent that longer glaciers have a larger area in the assessed glaciers (Figure 2A). The altitude of the terminus position varies from 4030 (Jumthul Glacier) to 5490 m asl. (Lonak Glacier) with a median value of 4950 m asl which divides the studied glaciers into two equal groups. The longer glaciers have a lower terminus position (Figure 2B) in the study area. The mean altitude of the glaciers ranges from 4935 to 6200 m asl with a median of 5622.5 m asl. Figure 2C indicates that lengthy glaciers have a higher mean altitude. South Lonak Glacier attains a maximum altitude up to 7200 m asl, while Gyamtang Glacier reaches up to 5500 m asl in the accumulation zone. The median value for the maximum altitude is 6250 m asl. Figure 2D depicts that the longer glaciers have higher maximum altitude in the selected glaciers. The slope gradient ranges from 115.2 (Changsang Glacier) to 449.7 m/km (Rula Glacier). The median slope gradient is 268.0 m/km. The longer glaciers have a lesser slope gradient as depicted in Figure 2E.

The glaciers with a simple form (11 glaciers), compound basin form (9 glaciers), and compound basins form (4 glaciers) have an average length of 5.0, 5.8, and 6.7 km, respectively (Table 1). Ablation zones of the two glaciers are oriented in E, seven glaciers in SE, six glaciers in S, four glaciers in SW, one in W and NW each, and three in NE (Figure 2F). The average length of the glaciers having an orientation in E, SE, S, SW, W, NW, and NE is 5.2, 6.2, 4.6, 5.4, 3.3, 6.6, and 6.9 km, respectively. Glaciers facing E to W orientation have a shorter length despite being located directly in the way of ascending monsoon. Out of 24 glaciers, 12 glaciers have glacial lakes. Proglacial lakes have been developed in front of glaciers such as Lonak, South Lonak, Tista, Tenbawa, and Jumthul. Changsang and Jongsang glaciers have supraglacial lakes too. The average length of the glaciers with no lake, proglacial lake, and supraglacial lake is 5.8, 4.8, and 8.0 km, respectively (Table 1). Figure 2 shows the scatterplots of the length of the glaciers vs. glacier area, bergschrund altitude, mean altitude, slope gradient, terminus altitude, and ablation orientation. It is apparent that areas of the glacier, bergschrund altitude, and mean altitude have a positive impact on the length of the selected glaciers. However, the impact of slope gradient and terminus altitude on the length of the glaciers is negative.

**Table 1:** Characteristics of medium-sized glaciers in Sikkim Himalaya (2000)

Name	Longitude (E)	Latitude (N)	Length (km)	Area (km^2^)	Max. Alt. (m)	Min. Alt. (m)	Sl. Gradi. (m/km)	Mean Alt. (m)	Orri. Abl.	Lakes	Form
Changme	88° 41' 2.173″	27° 57' 35.969″	5.9	4.6	5800	4810	169.2	5305	S	N	Simple
Changsang	88° 12' 37.314″	27° 48' 45.179″	8.7	11.3	6400	5400	115.2	5900	NE	S	Compound basin
Chuma	88° 32' 4.695″	27° 58' 13.588″	3.1	2.7	6000	4900	358.3	5450	S	N	Simple
E. Langpo	88° 13' 7.324″	27° 51' 25.505″	4.0	5.3	6400	5200	300.8	5800	SE	P	Simple
Gyamtang	88° 37' 22.591″	27° 49' 31.575″	3.3	2.4	5500	4370	341.4	4935	W	N	Simple
Jongsang	88° 11' 16.128″	27° 52' 24.328″	7.4	12.0	6900	5390	203.8	6145	NE	S	Compound basin
Jumthul	88° 20' 26.144″	27° 41' 56.204″	7.4	7.3	6300	4030	306.8	5165	SE	P	Compound basin
Kangkyong	88° 50' 27.224″	27° 53' 55.799″	8.0	20.8	6400	4450	242.8	5425	SW	N	Compound basin
Lonak	88° 8' 16.750″	27° 55' 2.766″	3.9	6.2	6400	5490	235.8	5945	E	P	Simple
N. Lonak	88° 9' 13.268″	27° 56' 25.796″	5.8	6.6	6200	5400	137.0	5800	SE	N	Simple
Onglaktang	88° 9' 49.748″	27° 35' 0.475″	7.0	7.3	6300	4380	274.7	5340	S	N	Compound basins
E. Rathong	88° 6' 49.707″	27° 35' 36.971″	7.8	5.9	6870	4660	282.6	5765	SE	N	Simple
Rula	88° 39' 40.971″	27° 53' 35.538″	3.8	3.2	6700	5000	449.7	5850	SE	N	Compound basin
S. Lonak	88° 9' 23.943″	27° 53' 57.794″	6.5	10.5	7200	5200	305.8	6200	E	P	Simple
S. Simpu	88° 14' 52.658″	27° 39' 32.649″	5.6	6.9	5800	4300	266.0	5050	S	N	Compound basins
Tasha	88° 31' 21.732″	28° 01' 6.012″	5.0	4.8	6200	5090	222.4	5645	SW	P	Compound basin
Tasha-1	88° 31' 9.266″	27° 59' 54.331″	3.2	2.5	6100	5450	205.7	5775	S	P	Simple
Tenbawa	88° 48' 15.359″	27° 55' 12.823″	5.1	5.9	6200	5180	199.2	5690	SW	P	Compound basin
Theukang	88° 19' 47.337″	27° 48' 49.562″	4.5	2.9	5900	4690	270.1	5295	NE	N	Simple
Tista	88° 49' 15.934″	27° 56' 55.252″	6.6	7.4	6800	5390	214.0	6095	NW	P	Simple
Toklung	88° 45' 48.292″	27° 50' 31.110″	3.1	2.2	5600	4700	294.1	5150	S	P	Compound basin
Tongshong	88° 12' 28.940″	27° 39' 17.794″	6.7	5.9	5800	4400	210.2	5100	SE	N	Compound basins
Umaram	88° 16' 44.792″	27° 42' 3.651″	7.6	7.9	6300	4050	296.4	5175	SE	N	Compound basins
Yulhe	88° 39' 31.886″	27° 58' 11.665″	3.5	2.9	6200	5000	340.9	5600	SW	P	Compound basin

Lakes: N- No lake; P- Proglacial Lake; S- Supraglacial Lake.

**Figure 3:**
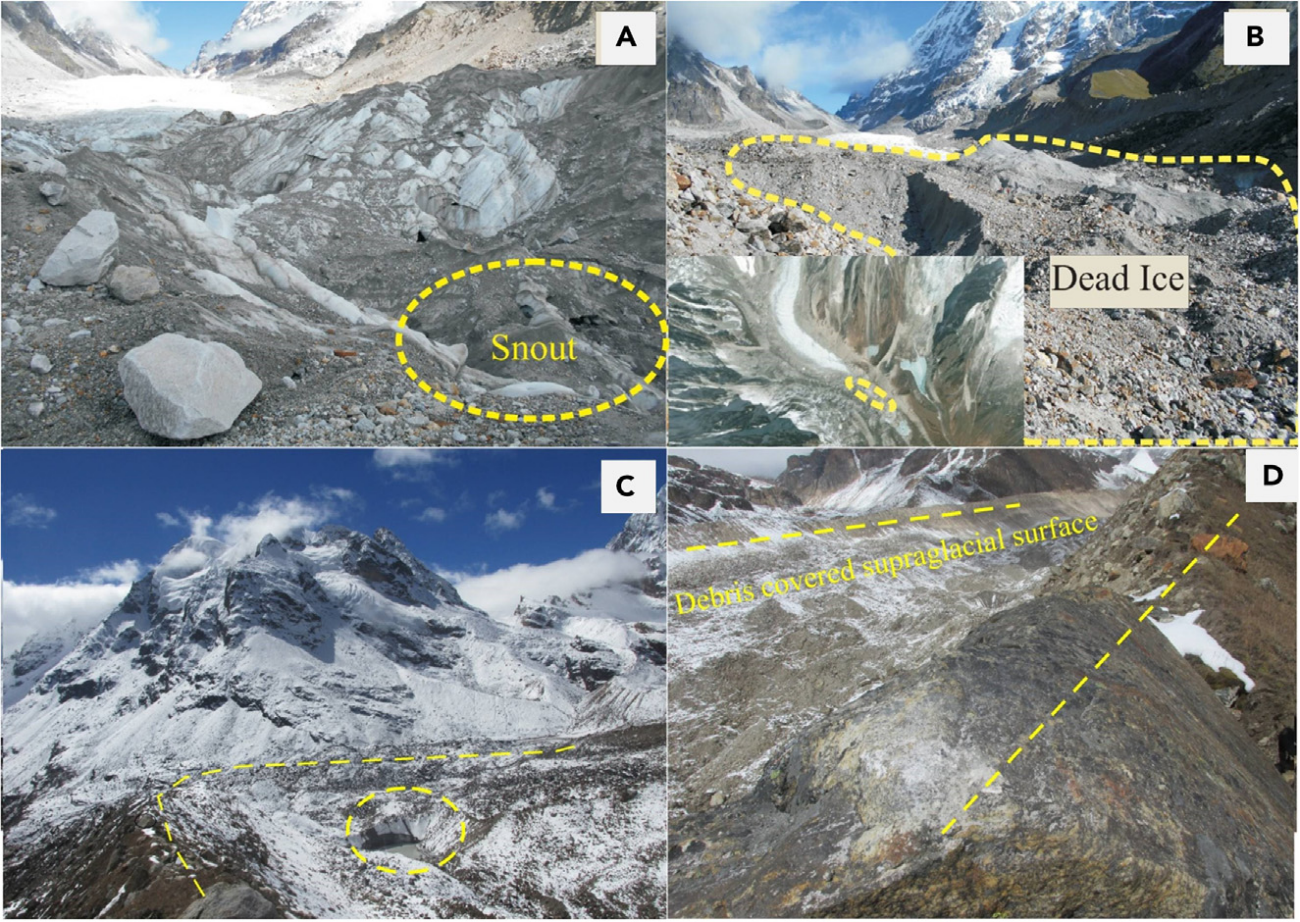
Field-study based inputs (A) GPS position of Snout (27^O^33′46.51″N, 88^O^6′50.44″E) of East Rathong Glacier on 13/06/2013. (B) Dead ice in front of East Rathong glacier snout (Google Earth Pro Image in inset). (C) Latero-frontal end moraines and a supraglacial lake on Changme glacier. (D) Lateral moraines and debris covered supraglacial surface of Changme glacier.

The snout of maximum glaciers is covered by thick debris cover. Figure 3 shows the snout and supraglacial surface of East Rathong and Changme glaciers which are covered by debris. The area in front of the East Rathong glacier is decaying at its place and seems to be stable for a long time as evident in Figure S1A and the same is also supported by field investigation (Figures 3A and 3B). Glaciers are confined within the well-preserved moraines landform (Figures 3C and 3D). An example of the formation of a supraglacial lake is marked in Figure 3C which shows the frontal area of Changme glacier.

### Terminus length fluctuations

Out of total 24 assessed glaciers, 12 glaciers did not retreat from 1988 to 2018. The remaining 12 glaciers have retreated at diverse rates during the observed period (Table 2). Since only half of the observed glaciers (12) have retreated, a glacier-wise description of the pattern of the retreat is presented here, which is also useful for comparison with glaciers of similar characteristics in other parts of the Himalayas. Among the 12 retreating glaciers (Chuma, Changsang, East Langpo, Jumthul, Lonak, Onglaktang, East Rathong, Rula, South Lonak, Tasha-1, Tista, and Tongshong), only seven glaciers (East Langpo, Lonak, South Lonak, East Rathong, Rula, Tista and Tasha-1) have retreated continuously from 1988 to 2018. Among these retreating glaciers, Chuma and Onglaktang glaciers retreated 115.4 ± 10.3 and 128.6 ± 11.5 m with rates of 3.9 and 4.3 m yr^−1^, respectively, which is the lowest during the entire assessed period from 1988 to 2018. At the same time, Changsang and S. Lonak glaciers showed a maximum retreat of 1917.1 ± 170.8 and 1328.8 ± 118.4 m with annual rates of 63.9 and 44.3 m yr^−1^, respectively. The maximum rate of retreat of Changsang and South Lonak glaciers is caused by their termination into the proglacial lakes (Figure S1C, and Table 1). The proglacial lake of Changsang Glacier is formed by the merging of existing supraglacial lakes together in later years. Other remaining glaciers i.e., East Langpo (465.2 ± 41.5 m, 15.5 m yr^−1^), Jumthul (217.2 ± 19.4 m, 7.2 m yr^−1^), Lonak (646.3 ± 57.6 m, 21.5 m yr^−1^), East Rathong (284.0 ± 25.3 m, 9.5 m yr^−1^), Rula (312.2 ± 27.8 m, 10.4 m yr^−1^), Tasha-1 (720.0 ± 64.2 m, 24.0 m yr^−1^), Tista (413.4 ± 36.8 m, 13.8 m yr^−1^), and Tongshong (147.3 ± 13.1 m, 4.9 m yr^−1^) have retreated at diverse rates during the entire assessed period (Table 2).

**Table 2:** Changes in glaciers terminus length (m) in Sikkim (1988–2018)

Name of Glaciers	Change 1988–2000	Annual Rate (1988–2000)	Change 2000–2009	Annual Rate (2000–2009)	Change 2009–2018	Annual Rate (2009–2018)	Total Change 1988–2018	Annual Rate (1988–2018)
Changme	0.0 (±0.0)	0.0	0.0 (±0.0)	0.0	0.0 (±0.0)	0.0	0.0 (±0.0)	0.0
Changsang	0.0 (±0.0)	0.0	1293.4 (±115.1)	143.7	623.7 (±55.5)	69.3	1917.1 (±170.8)	63.9
Chuma	81.6 (±7.3)	6.8	33.9 (±3.0)	3.8	0.0 (±0.0)	0.0	115.4 (±10.3)	3.9
E. Langpo	167.4 (±14.9)	14.0	154.3 (±13.7)	17.2	143.5 (±12.8)	15.9	465.2 (±41.5)	15.5
Gyamtang	0.0 (±0.0)	0.0	0.0 (±0.0)	0.0	0.0 (±0.0)	0.0	0.0 (±0.0)	0.0
Jongsang	0.0 (±0.0)	0.0	0.0 (±0.0)	0.0	0.0 (±0.0)	0.0	0.0 (±0.0)	0.0
Jumthul	0.0 (±0.0)	0.0	0.0 (±0.0)	0.0	217.2 (±19.3)	24.1	217.2 (±19.4)	7.2
Kangkyong	0.0 (±0.0)	0.0	0.0 (±0.0)	0.0	0.0 (±0.0)	0.0	0.0 (±0.0)	0.0
Lonak	128.3 (±11.4)	10.7	195.7 (±17.4)	21.7	322.3 (±28.7)	35.8	646.3 (±57.6)	21.5
N. Lonak	0.0 (±0.0)	0.0	0.0 (±0.0)	0.0	0.0 (±0.0)	0.0	0.0 (±0.0)	0.0
Onglaktang	128.6 (±11.5)	10.7	0.0 (±0.0)	0.0	0.0 (±0.0)	0.0	128.6 (±11.5)	4.3
E. Rathong	92.3 (±8.2)	7.7	67.1 (±6.0)	7.5	124.5 (±11.1)	13.8	284.0 (±25.3)	9.5
Rula	51.4 (±4.6)	4.3	85.3 (±7.6)	9.5	175.6 (±15.6)	19.5	312.2 (±27.8)	10.4
S. Lonak	404.2 (±36.0)	33.7	415.8 (±37.0)	46.2	508.8 (±45.3)	56.5	1328.8 (±118.4)	44.3
S. Simpu	0.0 (±0.0)	0.0	0.0 (±0.0)	0.0	0.0 (±0.0)	0.0	0.0 (±0.0)	0.0
Tasha	0.0 (±0.0)	0.0	0.0 (±0.0)	0.0	0.0 (±0.0)	0.0	0.0 (±0.0)	0.0
Tasha-1	317.9 (±28.3)	26.5	91.4 (±8.1)	10.2	310.7 (±27.7)	34.5	720.0 (±64.2)	24.0
Tenbawa	0.0 (±0.0)	0.0	0.0 (±0.0)	0.0	0.0 (±0.0)	0.0	0.0 (±0.0)	0.0
Theukang	0.0 (±0.0)	0.0	0.0 (±0.0)	0.0	0.0 (±0.0)	0.0	0.0 (±0.0)	0.0
Tista	245.2 (±21.8)	20.4	98.2 (±8.7)	10.9	70.1 (±6.2)	7.8	413.4 (±36.8)	13.8
Toklung	0.0 (±0.0)	0.0	0.0 (±0.0)	0.0	0.0 (±0.0)	0.0	0.0 (±0.0)	0.0
Tongshong	0.0 (±0.0)	0.0	0.0 (±0.0)	0.0	147.3 (±13.1)	16.4	147.3 (±13.1)	4.9
Umaram	0.0 (±0.0)	0.0	0.0 (±0.0)	0.0	0. 0 (±0.0)	0.0	0.0 (±0.0)	0.0
Yulhe	0.0 (±0.0)	0.0	0.0 (±0.0)	0.0	0.0 (±0.0)	0.0	0.0 (±0.0)	0.0

Temporally, the retreating glaciers have fluctuated with heterogeneous patterns during the different periods. Out of 12 retreating glaciers, only 9 glaciers retreated with varied rates between 1988 and 2000 as Changsang, Jumthul, and Tongshong glaciers did not show any retreat. During this period, the maximum and minimum retreat was recorded at the snout of South Lonak (404.2 ± 36.0 m, 33.7 m yr^−1^) and Rula (51.4 ± 4.6 m, 4.3 m yr^−1^) glaciers, respectively. In the next time band between 2000 and 2009, out of previously 9 retreating glaciers, only 8 glaciers have recorded a retreat at their snout. One glacier (Onglaktang) did not retreat during 2000–2009 and for the further assessed period as well. However, Changsang Glacier started retreating afresh during this period and recorded a maximum retreat of 1293.4 ± 115.1 m (143.7 m yr^−1^) which is also the highest rate of retreat for the entire assessed period. Chuma glacier has recorded the minimum retreat (33.9 ± 3.0 m, 3.8 m yr^−1^) during the same period. In the recent period between 2009 and 2018, Chuma glacier has stopped retreating, but two more glaciers (Jumthul and Tongshong) started retreating afresh which has increased the number of retreating glaciers to 10. The maximum retreat was recorded at the snout of Changsang Glacier (623.7 ± 55.5, 69.3 m yr^−1^). Whereas, Tista Glacier recorded the minimum retreat (70.1 ± 6.2 m, 7.8 m yr^−1^) during 2009–2018. The rate of the retreat accelerated during the recently observed span between 2009 and 2018.

The reported frontal retreat has caused a total area loss of 3.95 (±0.35) km^2^ with a rate of 0.13 km^2^ yr^−1^ (2.53%, 0.08% yr^−1^). The total areal coverage of 24 glaciers in Sikkim was 156.12 (±12.44) km^2^ in 1988 which has reduced to 152.17 (±6.07) km^2^ in 2018. However, in reference to the total area of only 12 retreating glaciers (75.42 km^2^), the total loss of the area is 5.23% from 1988 to 2018.

## Discussion

The study attempts to understand the frontal retreat of the medium-sized glaciers in Sikkim Himalaya. The heterogeneity of the glacier’s retreat may be understood in the purview of climatic and non-climatic factors in the region. Intra-regional heterogeneity in the retreating pattern may be caused by topographical factors if glaciers are located in similar climatic settings as in the case of the present glaciers in Sikkim Himalaya. Whereas, the inter-regional disparity in the retreating pattern of the glaciers may be caused by prevailing different climatic conditions in the respective regions. Therefore, the following section discusses the possible causes for the intra-regional and inter-regional heterogeneous fluctuation of termini of glaciers.

### Topographical factors and terminus retreat

**Figure 4:**
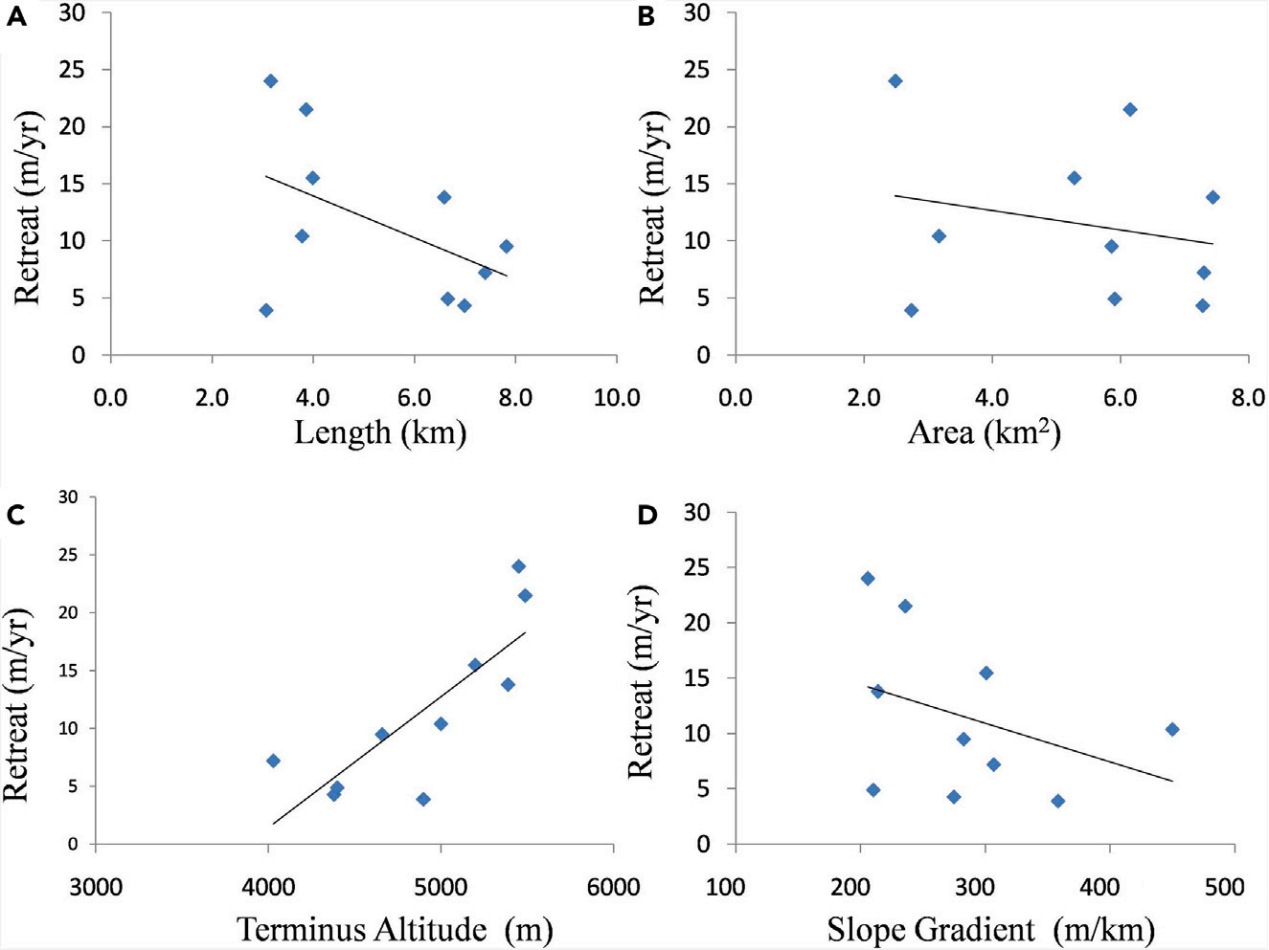
Relationship between the rate of retreat of glacier terminus with topographical factors Rate of retreat vs. (A) glacier length, (B) glacier area, (C) terminus altitude, and (D) slope gradient.

The terminus of assessed glaciers showed a heterogeneous pattern of retreat even being located in the same basin which has largely similar climatic conditions. The heterogeneity in the fluctuation of terminus can be understood by investigating the impact of topographical factors.^15^^,^^18^ Since Changsang (63.9 m yr^−1^) and S. Lonak (44.3 m yr^−1^) glaciers recorded exceptionally higher rates of retreat probably due to the existence of glacial lakes at their snouts, both the glaciers are considered outliers as identified by the Grubbs test (p value - 0.001, alpha value - 0.05).^43^ Therefore, both glaciers are omitted to understand the retreat rate in reference to the topographical factors in a better way for further analysis. Figure 4 shows the scatterplots of glacier retreat rate vs. glacier area, length, terminus altitude, and slope gradient in Sikkim Himalaya. It is apparent that glaciers with shorter lengths and smaller areas have retreated at faster rate (Figures 4A and 4B). It is also observed that glaciers with higher terminus altitudes and lesser slope gradients have retreated at faster rates (Figures 4C and 4D). Other topographical factors such as the form of a glacier and orientation of the ablation zone of the glaciers depict a control over the retreating pattern of the glaciers in the region. In respect to the form of the glaciers, four glaciers are categorized as compound basins among which only two glaciers retreated at the rate of 4.3 and 4.9 m yr^−1^. The eight glaciers possessed the form of a compound basin, out of them only two glaciers retreated at the rate of 7.2 and 10.4 m yr^−1^. Ten glaciers were of simple form among which 6 glaciers have receded the most in a range from 3.9 to 24.0 m yr^−1^. Orientation-wise, the ablation zone of glaciers is oriented in different directions. Two glaciers are oriented in NE and none of them have retreated. One glacier, oriented in E has retreated at 21.5 m yr^−1^. Out of seven glaciers that are oriented in SE, five glaciers have retreated in a range of 4.9 m to 15.5 m yr^−1^. Six glaciers are oriented in S, out of which only three glaciers have receded with a range from 3.9 to 24.0 m yr^−1^. Four glaciers are oriented in SW and one glacier oriented in W has not retreated over the observed period. A glacier having ablation zone orientation in NW showed a retreat of 13.8 m yr-^1^.

Out of the 22 glaciers, 12 glaciers do not have any lake among which only 5 glaciers have retreated with a range from 3.9 to 10.4 m yr^−1^. Proglacial lakes exist in front of the 9 glaciers among which only 5 glaciers retreated with a range from 7.2 to 24 m yr^−1^. The continuous rate of retreat of Lonak and Tasha-1 glaciers is caused by the existence of proglacial lakes. It is evident from the previously mentioned analysis of medium-sized glaciers that glaciers with a shorter length, smaller area, higher terminus altitude, lower slope gradient, with a simple form, having proglacial lakes and oriented in E, SE, and S have retreated most over the entire observed period in the Sikkim Himalaya.

### Comparison of the retreat of terminus across the Himalayas

**Figure 5:**
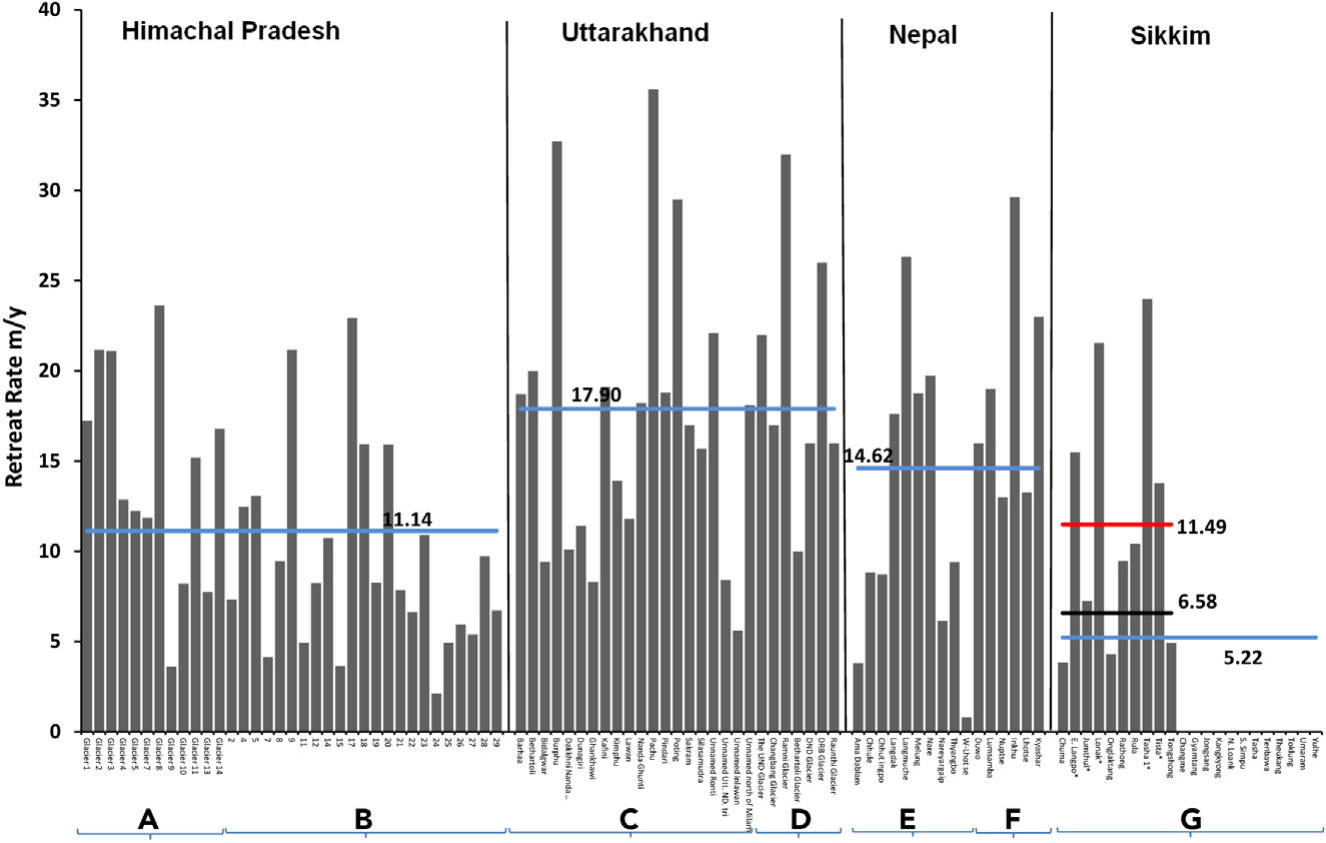
Rate of the retreat of the terminus of similar-sized glaciers across the Himalayas (A) Chandra-Bhaga basin,^28^ (B) Miyar basin,^47^ (C) Central Himalaya,^45^ (D) Central Himalaya,^46^ (E) South slope of Mt. Everest (central Southern Himalaya),^29^ (F) Mount Everest region, Nepal,^44^ (G) Present study. Glacier name with a star indicates a glacial lake at the snout of a glacier.

The present study shows that glaciers are retreating at a diverse rate in Sikkim. The retreating rate of the receding glaciers ranges from a minimum of 3.9 m yr^−1^ (Chuma glacier) to a maximum of 24.0 m yr^−1^ (Tasha-1 glacier). Since all the glaciers are medium-sized, the average retreat rate of receding glaciers could be a good indicator of terminus fluctuation for the whole Sikkim region. The average retreat rate of glaciers (only for retreating glaciers) in Sikkim is 6.58 m yr^−1^ after excluding the glaciers with proglacial lakes and 11.49 m yr^−1^ including the glaciers having proglacial lakes. Moreover, after considering the stagnant and retreating glaciers together, the average retreating rate is 5.22 m yr^−1^. A comparison of satellite image-based retreat rate of similar-sized glaciers with similar reporting methods reveals that glaciers in Nepal, Himachal, and Uttarakhand have retreated at higher rates (Figure 5). Glaciers in Nepal have retreated at an average rate of 14.62 m yr^−1^.^29^^,^^44^ In the case of Uttarakhand, glaciers have recorded an average rate of retreat of 17.90 m yr^−1^ ^45^^,^^46^ which is also higher than the retreat rate of glaciers in Sikkim Himalaya. Similarly, in Himachal Himalaya, glaciers have retreated at an average rate of 11.14 m yr^−1^.^28^^,^^47^ A previously published study also observed that some of the highest rates of retreat exist in the western Himalaya whereas glaciers in the southern central Himalaya are retreating at slower rates.^23^ Moreover, the rate of mass loss of glaciers also reveals a declining gradient from west to east over Spiti Lahaul, West Nepal, Everest, and Bhutan.^48^

### Climatic synthesis and inferences for glacier retreat in the Himalaya

After comparing the glaciers in Sikkim with other parts of the Himalayas, it appears that glaciers in Sikkim have retreated at a lower rate. The lower rate of retreat of glaciers in Sikkim may be explained by the regional climatic synthesis as discussed in the published studies on climatic variability in the Himalayan region.^41^ Broadly, Himalayan glaciers are dependent on the Indian summer monsoon and westerlies which have decreasing intensity from east to west and west to east, respectively. These climatic systems not only provide moisture for the glaciers but also control the temperature variation on spatial and temporal scales in the Himalayan region. In the recent past, several scholars have attempted to understand the changing nature of climate which is helpful to understand the complex dynamics of the glaciers in the region. The existing research on precipitation shows a complex trend in the Himalayan region.^41^ An earlier study on the northwestern Himalayas analyzed the precipitation data and found a statistically insignificant positive trend (at 95% confidence level) in winter precipitation and a statistically significant (95% level of confidence) negative trend in monsoon and overall annual precipitation between 1866 and 2006.^49^ In Uttarakhand, precipitation variability was investigated at 30 stations during 1902–1980 and it was found that while precipitation increased during the period 1902–1964, it showed a reversal during 1965–1980.^50^ Likewise, precipitation in the Kashmir valley recorded a decreasing trend in mountainous regions with the highest rate of decrease during 1980–2014.^51^ In the case of Nepal, a mixed trend in precipitation in the Kaligandaki river basin was reported as only two stations showed an increasing trend, while the remaining six stations exhibited no trends and none have recorded a negative trend in the annual precipitation.^52^ In the Northeast region of India, the annual precipitation values do not show any significant rise during 1971–2005.^53^ Similar results were documented in a study on the Northeast region as a whole highlighting that there is no clear trend in rainfall, although there are seasonal trends for some seasons and some hydro-meteorological subdivisions during 1871–2008.^54^

On part of the temperature, the existing studies on the Himalayan region confirm the warming trends at a diverse rate. A study on the northwestern Himalayas has reported on the temperature changes with one of the longest duration (1901–1989/2002) of data and recorded a significant rise of ∼1.6°C in temperature in the last century, with accelerated warming in winters.^55^ The same study also suggested that the maximum temperature has recorded a higher rate of increase than the minimum temperature in the northwestern Himalayas with noticeable warming occurring after the late 1960s as the last two decades experienced the highest rate of increase in warming.^55^ Similar results were also confirmed by another study on the western Himalaya which highlights that seasonal mean, maximum, and minimum temperatures have increased by ∼2°C, ∼2.8°C, and ∼1°C, respectively from 1984/85 to 2007/08.^56^ In the Kashmir valley too, trend analysis of annual mean temperature revealed a significant increase from 1980 to 2014 with accelerated warming during 1980–2014 and an increased rate in recent years (2001–2014).^51^ The same study also reported a steeper increase in the annual mean maximum temperature than the annual mean minimum temperature during 1980–2014.

In the central Himalayan region, the average warming occurred at 0.38°C per decade for three decades from 1980 to 2009, with a greater magnitude of warming in maximum temperature (0.65°C per decade) than minimum temperature (0.11°C per decade),^57^ similar to the northwestern Himalaya. The higher trend of maximum temperature and no change in the minimum temperature in Nepal is also confirmed by a study focused on the Kaligandaki river basin, Himalaya, Nepal.^52^

In the case of the eastern Himalayas and northeastern region of India, similar average warming has been reported but with reverse characteristics of maximum and minimum temperatures.^53^^,^^54^^,^^58^^,^^59^ A study conducted on the Northeast region of India analyzed climatic data of 8 stations from the 1970s/1980s to 2000 and has reported average warming in a range of 0.2°C–0.8°C per decade at four stations which is almost similar to the northwestern and central Himalayas.^58^ However, the same study found that the minimum temperature has increased with a range of 0.1°C–0.6°C per decade at the majority of stations, and no trend was found in the maximum temperature at the majority of observed stations. Another study on Northeast India also confirmed similar characteristics of minimum and maximum temperatures by reporting that the annual minimum temperature increased by 0.04°C per year which resulted in a total of 2.1°C increase and the annual maximum temperature does not show any systematic trend in the region during the period 1971–2005.^53^ An earlier study also reported a negative slope for the maximum temperature and no slope for the minimum temperature for the northeast region during 1951–2003.^54^ In the case of Sikkim Himalaya, the average temperature recorded a positive trend at Tadong (1.05°C) and Gangtok (1.98‬˚C) from 1980 to 2010 along with positive trends in the minimum temperatures (Gangtok: 2.05°C and Tadong: 1.95°C) and no or negative trend in the case of maximum temperature at both observatories.^41^ In brief, the warming in the average temperature in Sikkim Himalayas is almost similar to the warming in the northwestern Himalayas. However, contrary trends in the case of both minimum as well as maximum temperatures are noticed over northwest-central Himalaya and eastern Himalaya.

The reported similar average warming in the northwest, central, and eastern Himalayas does not help much in explaining the lower rate of retreat of glaciers in Sikkim. Precipitation also does not have any particular trend in the Himalayan region. However, the reported contrary trends in the case of the minimum and maximum temperatures in the eastern Himalaya along with Sikkim and other parts of the Himalayas may be helpful to explain the lower retreat rates of the glacier in Sikkim. A recent study on climatic variability in the region and the references therein have explained the possible reason behind such contrary trends and suggested that different precipitation regimes are responsible for the same.^41^ The relatively clear sky during the summer season may cause the maximum temperature to shoot up and may have resulted in a relatively higher melting of glaciers in the northwestern and central Himalayas. However, the eastern Himalayas (present study area) experiences extensive cloud cover and heavy precipitation^60^ due to the dominance of the Bay of Bengal branch of Indian summer monsoon which controls maximum temperature during the summer season and may have resulted in a lower rate of retreat of glaciers in Sikkim Himalayas. Therefore, it may be inferred that the disparity in the retreat rate of glaciers in Sikkim and other parts of the Himalayas is caused by the differential summer melting and associated responsible factors. The trend in the retreat rate of glaciers from Sikkim to Uttarakhand may be attributed to the decreasing intensity of the Indian summer monsoon from east to west. The relatively lesser retreat rate in Himachal Pradesh may be caused by the impact of moisture carried by westerlies.

### Conclusion

The study presents spatial and temporal changes at the termini of medium-sized glaciers in the Sikkim Himalaya. The retreat rate ranges from 63.9 to 3.9 m yr^−1^ with a total areal change of 3.95 ± 0.35 km^2^ (2.53%, 0.08% yr^−1^) during 1988–2018. Only 12 glaciers recorded fluctuations at their terminus at varied rates during the observed period. The retreat rate was accelerated during the last decades. The topographical factors seem to have control on the intra-regional pattern of retreat in the region as glaciers with a shorter length, smaller area, higher terminus altitude, lesser slope gradient, with a simple form, having lakes and oriented in E, SE, and S have retreated the most over the entire observation period in the Sikkim Himalaya. Supraglacial lakes, if developed and merged, could cause higher deglaciation in the region. An inter-regional pattern of the retreat is explored by comparing the Sikkim glaciers with glaciers of other parts of the Himalayas which reveals that glaciers in Sikkim are retreating at a lower rate than in other parts of the Himalayas. The lower rate of recession of glaciers in Sikkim may be explained by differential summer melting in the Himalayan region which is controlled by prevailing precipitation regimes in the Himalayas.

## STAR★Methods

### Key resources table

**Table:** 

REAGENT or RESOURCE	SOURCE	IDENTIFIER
**Deposited data**		

Landsat TM, Accq. Date: 01/12/1988 (Spatial Resolution: 30 m)	https://earthexplorer.usgs.gov/	LT51390411988336BKT00
Landsat ETM+, Accq. Date: 26/12/2000 (Spatial Resolution: 15–30 m)	https://earthexplorer.usgs.gov/	LE71390412000361SGS00
Landsat TM, Accq. Date: 10/02/2009 (Spatial Resolution: 30 m)	https://earthexplorer.usgs.gov/	LT51390412009041KHC00
Landsat OLI, Accq. Date: 18/01/2018 (Spatial Resolution: 15–30 m)	https://glovis.usgs.gov/	LC81390412018018LGN00

### Resource availability

#### Lead contact

Any request for further information and resources should be directed to the lead contact Parvendra Kumar at parvendra.jnu@gmail.com.

#### Materials availability

All the used recourses are provided in the key resources table.

### Method detail

The present study is based on remotely sensed datasets along with the aid of primary field investigations. With synoptic and repetitive characteristics, remote sensing has emerged as a robust method to study the rugged and inaccessible terrains of the Himalayas. However, at the same time, ground verification by the primary survey is an essential part of any scientific study and therefore has been carried out in the study area for the limited glaciers.

#### Satellite images

Satellite images of the Landsat Thematic Mapper (1988 & 2009), Enhanced Thematic Mapper Plus (2000), and Operational Land Imager (2018) have been used to demarcate the boundaries and other parameters of glaciers for the respective years. Additionally, the digital elevation model (DEM) i.e., Advanced Spaceborne Thermal Emission and Reflection Radiometer (ASTER) Global Digital Elevation Model Version 2 (ASTER GDEMV2) is used to quantify the glacier’s topographical characteristics. All the satellite images are acquired from the websites of the United States Geological Survey. All the selected images are with less than 10% cloud cover and have minimum seasonal snow. The terminus of the observed glaciers was not having any significant snow cover in all the images. Details of the used satellite images are given in the Key Resource Table. The Landsat Thematic Mapper (TM) has seven spectral bands, including bands in shortwave IR and thermal IR with a spatial resolution of 30–120 m (thermal).^61^^,^^62^ The Enhanced Thematic Mapper Plus (ETM+) provides multispectral coverage similar to TM but with a 60 m thermal band and a new 15 m panchromatic band.^62^^,^^63^ In addition to six refined heritage bands ranging from blue to SWIR, the Landsat 8 (OLI) provides three new bands with improved signals for a better understanding of land cover.^64^^,^^65^ After a prevalidation study, the joint ASTER mission of the National Aeronautics and Space Administration (NASA) and the Ministry of Economy, Trade and Industry (METI) introduced the revised DEM data product (ASTER GDM V2) in 2011 with average vertical accuracy within −0.2 m when compared to ∼18000 geodetic reference points and an accuracy of 17 m at the 95% confidence level.^66^ For the present study, ASTER GDEM V2 (1^o^x1°) with a spatial resolution of 30 m data is used (ASTGTM2_N27E088 & N28E088).

All the selected images of LANDSAT used in this study are ortho-rectified with the processing of Level 1 Precision and Terrain corrected products (L1TP) with RMS Error of <12m. The L1TP data contains the highest quality and is considered suitable for time-series analysis.^67^ All the LANDSAT images are in the same coordinate system of WGS 1984 UTM Zone 45N.

#### Glacier mapping and change assessment

The delineation of glaciers boundary and classification of glaciers is based on the information drawn from the Global Land Ice Measurements from Space (GLIMS) manual.^68^^,^^69^ All glacier boundaries are delineated manually on-screen by digitizing methods based on visual interpretation with false-color composite (FCC) developed from multispectral bands of satellite images.^29^ Besides, Normalized Difference Snow Index (NDSI) and band rationing methods have been helpful to identify boundaries of clean snow and ice in accumulation zones.^70^^,^^71^^,^^72^ However, glacier mapping in debris-covered ablation zones was a difficult exercise for the present study area as NDSI and band ratio produced similar results for the debris-covered part of the glaciers and adjoining barren land due to the same reflectance.^73^ Therefore, the delineation of glacier boundaries in the present study is majorly carried out manually. Additionally, temporal images of Google Earth Pro with 3D views and ESRI online base maps in ArcMap also have been used in glacier mapping.

The delineation of the glacier area is based on the identification of the glacial morphological signature, such as lateral moraines, dead-ice in the frontal part of the glaciers (Figure S1A), bergschrund (from Google Earth 3D view and ArcMap base map) (Figure S1B), glacial lakes (Figure S1C) and other associated glacial landforms. The probable location of a snout is also identified based on a stream-line.^74^ The GPS-based field investigation has been helpful to mark the location of snouts more accurately in some cases. We further compared snout positions with the associated geomorphic features of glaciers on the Google Earth images. The change in the snout position of a glacier is calculated by drawing parallel lines at intervals of 50 m on the glacier’s outline and calculating their average (Figure S1C).^29^

The Landsat ETM+ image (2000) is used as a baseline dataset for glacier mapping. The Landsat TM images are utilized to demarcate the glacial boundaries for the years 1988 and 2009. The Landsat OLI (2018) data is used to map the latest glacier outlines of 2018. The multispectral bands of the ETM+ (2000) and OLI (2018) images have been Pan-sharpened with the higher spatial resolution panchromatic band to enhance the spatial resolution from 30 m to 15 m through a Brovey transform image fusion technique.^18^ These Pan-sharpened images were helpful for the accurate identification of the glacier’s terminus and associated features relatively.

#### Measurement of uncertainty in Glacier mapping

The positional accuracy of the used images comes under acceptable limits. The overlapping of images revealed a good match too. Therefore, the error in positional accuracy is considered negligible for this study. However, measurement of uncertainty is a necessary step in glacier mapping due to the medium spatial resolution of used geospatial datasets and the presence of debris cover over an ablation zone of the glaciers. Hence, the mapping uncertainty is assessed by buffering the area of glaciers by one pixel around the perimeter of each glacier boundary.^2^ It produced an error of ±7.97%, ±3.98%, ±7.96% and ±3.98% of the total area of glaciers for 1988, 2000, 2009 and 2018, respectively. The uncertainty in a measurement of fluctuation of the length of a glacier between the two years is calculated with the following equation. In the equation, ‘e’ is the calculated uncertainty in the mapping of glaciers through the buffering method for a particular year.

(Equation 1) $$E_{1988-2000}=\sqrt{e_{1988}^{2}+e_{2000}^{2}}$$

#### Field work

Field visits were conducted to East Rathong and Changme glaciers during the years 2013 and 2016. The terminus’s positions of the same glaciers were demarcated with Garmin GPS Map 76, 78. Photographic evidence was collected to observe the geomorphic aspects of the glacial valleys which may hold control on the behavior of the glaciers. Usually, these details are not possible to capture with remotely sensed images. These field photographs of glacial geomorphic information are immensely helpful in understanding the complex aspects related to glacier characteristics.

## Data Availability

All the generated data is included in the article. No code is generated in this study.
