# Supplementary material for: Frontal changes in medium-sized glaciers in Sikkim, India during 1988–2018: Insights for glacier-climate synthesis over the Himalaya
Source: iScience. 2023 Aug 30;26(10):107789. doi: 10.1016/j.isci.2023.107789 (PMC10514448; doi:10.1016/j.isci.2023.107789)
Supplement: Document S1. Figures S1 [file mmc1.pdf]

iScience, Volume *26*

Supplemental information

Frontal changes in medium-sized glaciers in Sikkim, India during 1988–2018: Insights for glacier-climate synthesis over the Himalaya

Parvendra Kumar and Milap Chand Sharma

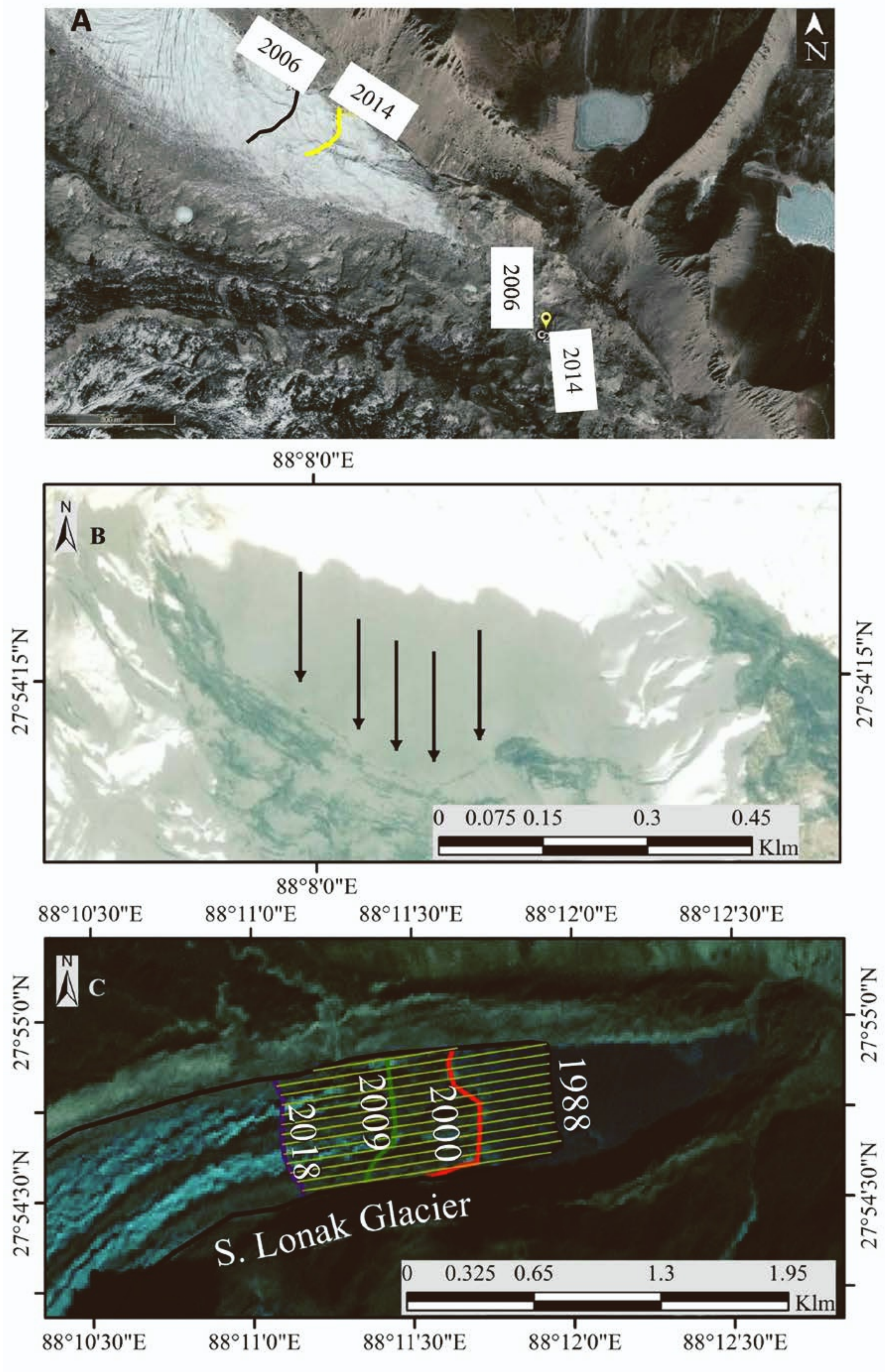

Figure S1: A- Relative ice movement on East Rathong Glacier and dead ice in front through multi-temporal images from Google Earth Pro, B -Visibility and demarcation of bergschrund on ArcMap base map for marking the glacier boundary, C- Measurement of the retreat of a glacier's snout (ETM+ 2000 pan-sharpen image in background) and S. Lonak proglacial lake.
